# Secreted PD-L1 variants mediate resistance to PD-L1 blockade therapy in non–small cell lung cancer

**DOI:** 10.1084/jem.20180870

**Published:** 2019-03-14

**Authors:** Bo Gong, Kazuma Kiyotani, Seiji Sakata, Seiji Nagano, Shun Kumehara, Satoko Baba, Benjamin Besse, Noriko Yanagitani, Luc Friboulet, Makoto Nishio, Kengo Takeuchi, Hiroshi Kawamoto, Naoya Fujita, Ryohei Katayama

**Affiliations:** 1 Cancer Chemotherapy Center, Japanese Foundation for Cancer Research, Tokyo, Japan; 2 Department of Computational Biology and Medical Sciences, Graduate School of Frontier Sciences, The University of Tokyo, Chiba, Japan; 3 Immunopharmacogenomics Group, Cancer Precision Medicine Center, Japanese Foundation for Cancer Research, Tokyo, Japan; 4 Pathology Project for Molecular Targets, Cancer Institute, Japanese Foundation for Cancer Research, Tokyo, Japan; 5 Laboratory of Immunology, Institute for Frontier Life and Medical Sciences, Kyoto University, Kyoto, Japan; 6 Department of Hematology and Oncology, Graduate School of Medicine, Kyoto University, Kyoto, Japan; 7 INSERM U981, Gustave Roussy Cancer Campus, Université Paris Saclay, Villejuif, France; 8 Department of Cancer Medicine, Gustave Roussy Cancer Campus, Villejuif, France; 9 The Cancer Institute Hospital, Japanese Foundation for Cancer Research, Tokyo, Japan; 10 Division of Pathology, Cancer Institute, Japanese Foundation for Cancer Research, Tokyo, Japan

## Abstract

Immune checkpoint blockade against programmed cell death 1 (PD-1) and its ligand PD-L1 often induces durable tumor responses in various cancers, including non–small cell lung cancer (NSCLC). However, therapeutic resistance is increasingly observed, and the mechanisms underlying anti–PD-L1 (aPD-L1) antibody treatment have not been clarified yet. Here, we identified two unique secreted PD-L1 splicing variants, which lacked the transmembrane domain, from aPD-L1–resistant NSCLC patients. These secreted PD-L1 variants worked as “decoys” of aPD-L1 antibody in the HLA-matched coculture system of iPSC-derived CD8 T cells and cancer cells. Importantly, mixing only 1% MC38 cells with secreted PD-L1 variants and 99% of cells that expressed wild-type PD-L1 induced resistance to PD-L1 blockade in the MC38 syngeneic xenograft model. Moreover, anti–PD-1 (aPD-1) antibody treatment overcame the resistance mediated by the secreted PD-L1 variants. Collectively, our results elucidated a novel resistant mechanism of PD-L1 blockade antibody mediated by secreted PD-L1 variants.

## Abstract (precis)

Therapeutic resistance to PD-L1 blockade therapy following an initial positive response is increasingly observed. Gong et al. show that secreted PD-L1 splicing variants act as “decoys,” mediating resistance to the PD-L1 blockade therapy.

## Graphical Abstract

**Figure:**
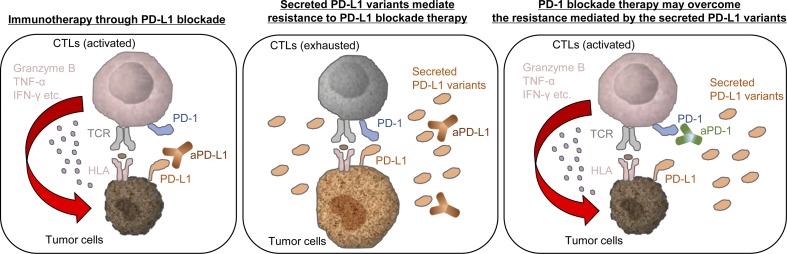

## Introduction

Programmed death ligand 1 (PD-L1), a member of the B7 family, is a putative type I transmembrane protein of 290 amino acids consisting of an IgV-like domain, an IgC-like domain, a transmembrane domain, and a cytoplasmic tail of 30 amino acids (Shi et al., 2013). PD-L1 is expressed on the surfaces of various cell types, including macrophages, dendritic cells, and endothelial cells in the heart (Shi et al., 2013). When PD-L1 interacts with its receptor on activated cytotoxic T cells, programmed cell death 1 (PD-1), via the IgV domain, PD-1 transiently forms negative costimulatory microclusters with TCRs and costimulatory receptor CD28 by recruiting phosphatase Src homology 2 domain-containing tyrosine phosphatase 2 (SHP2), leading to its dephosphorylation (Yokosuka et al., 2012; Hui et al., 2017). This results in effector T cell exhaustion by decreasing the phosphorylation of various signaling molecules such as ERK, Vav, and PLCγ, which regulate T cell activation and proliferation via the nuclear factor of activated T cells (NFAT; Yokosuka et al., 2012; Hui et al., 2017). PD-L1 is also abundantly expressed in various carcinoma cells such as lung, colon, melanoma, and leukemic cells and is involved in immune escape through its interaction with PD-1 (Shi et al., 2013; Ohaegbulam et al., 2015). Over the past decade, blockades of the PD-L1/PD-1 axis showed remarkable clinical response in a variety of advanced cancers (Yarchoan et al., 2017). However, clinical benefits have been observed in only 20–30% of patients in whom biomarkers for predicting the response are still to be identified (Callahan et al., 2016; Yarchoan et al., 2017). Recent studies have suggested that the high tumor mutation burden and CD28 expression in exhausted CD8 T cells predict the response to immune checkpoint inhibitors (Hui et al., 2017; Yarchoan et al., 2017). Moreover, the expression of PD-L1 in the tumor environment is suggested to be a biomarker of PD-1 blockade, because progression free survival significantly improved in patients with a PD-L1 expression level of ≥50% (Reck et al., 2016). Cytokines, such as IFN-γ, released from cytotoxic lymphocytes have been suggested to up-regulate PD-L1 expression (Garcia-Diaz et al., 2017). Furthermore, the structure alteration of the PD-L1 3′-untranslated region resulting in aberrant expression of PD-L1 in various cancers, including adult T cell leukemia/lymphoma, diffuse large B cell lymphoma, and stomach adenocarcinoma, may also allow cancer cells to escape the immune response. (Kataoka et al., 2016). Conversely, some studies associated soluble PD-L1 levels in patient plasma with better response to immune checkpoint inhibitors, particularly to anti–PD-1 (aPD-1) and anti–CTLA-4 antibodies in patients with melanoma or multiple myeloma (Wang et al., 2015; Zhou et al., 2017).

Non–small cell lung cancer (NSCLC) harbors a relatively high mutational landscape, and high tumor mutation burden tends to correlate with clinical benefits of PD-L1/PD-1 blockade treatments (Lawrence et al., 2013; Yarchoan et al., 2017). aPD-1/PD-L1 therapy is becoming a primary treatment option for patients with NSCLC (Robert et al., 2015; Reck et al., 2016). However, therapeutic resistance after initial response limits its effectiveness. Multiple mechanisms have been shown to be associated with acquired and primary resistance to aPD-1 therapy, including loss-of-function mutations in Janus kinases *JAK1/2*, truncating mutations or homozygous loss of β-2-microglobulin (*Β2M*), and lacking the allele-specific *HLA* or *PTEN* (Zaretsky et al., 2016; George et al., 2017; McGranahan et al., 2017; Shin et al., 2017). It was also suggested that expressing other inhibitory immune checkpoint molecules, such as T cell immunoglobulin domain and mucin domain-3 (TIM-3) and T cell immunoreceptor with Ig and ITIM domains (TIGIT) on tumor-infiltrated cytotoxic lymphocytes, or recruiting immunosuppressive cells such as regulatory T cells promoted PD-1 blockade resistance (Koyama et al., 2016; Sharma et al., 2017; Hung et al., 2018); however, the mechanisms of resistance to anti–PD-L1 (aPD-L1) therapies are mostly unknown.

In this study, we identified two unique secreted PD-L1 (sPD-L1) splicing variants lacking the transmembrane domain from two NSCLC patients who failed to respond to aPD-L1 treatment. From the additional RNA sequencing (RNA-seq) analysis conducted with post-treatment specimens obtained from 15 patients who were refractory to PD-L1 blockade therapy, we further found that two patients harbored the same sPD-L1 splicing variants. These sPD-L1 variants competitively interrupted the neutralizing activity of aPD-L1 antibody in vitro and induced resistance to aPD-L1 therapy in a MC38 syngeneic mouse model. More importantly, we demonstrated PD-L1 blockade resistance in vivo with a mixture of just 1% MC38 cells with sPD-L1 variant and 99% of cells that overexpressed wild type of PD-L1; this resulted from an accumulation of soluble PD-L1 in plasma. Consistent with this observation, the levels of soluble PD-L1 in the plasma or pleural effusion in patients detected with sPD-L1 splice variants were much higher than in healthy donors or patients without the sPD-L1 variants. Furthermore, PD-1 blockade antibody retained its inhibitory activity in the presence of sPD-L1 variants and was suggested to overcome the sPD-L1 variant–induced resistance in vitro and in vivo. Taken together, our data demonstrated that sPD-L1 splicing variants induced the resistance to PD-L1 blockade therapy by acting as a “decoy,” and aPD-1 treatment might be a therapeutic option for patients with sPD-L1 splicing variants.

## Results

### Identification of PD-L1 C-terminal–deficient splicing variants from a relapsed lesion to PD-L1 blockade in patients with NSCLC

**Figure 1.**
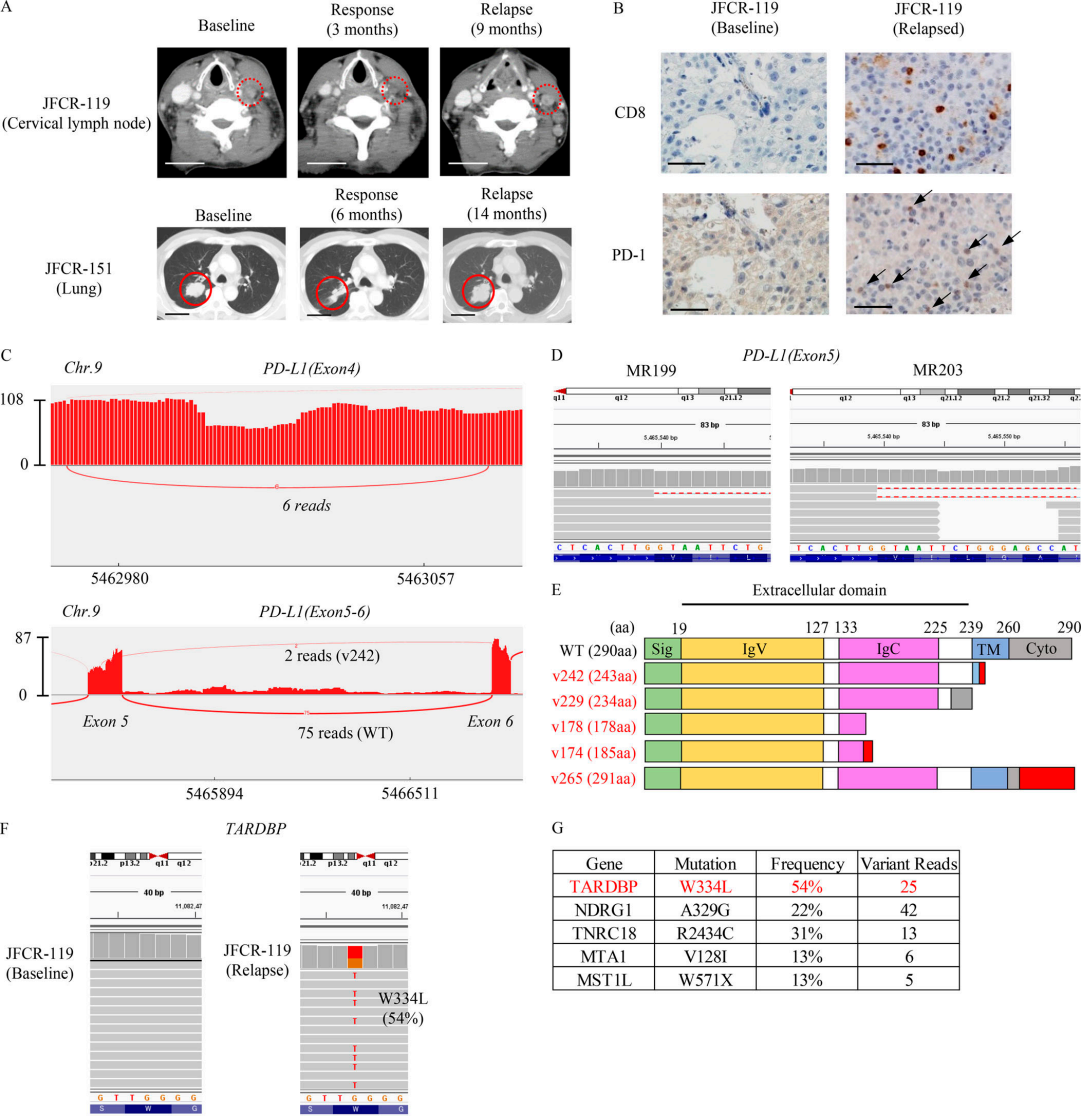
**Identification of PD-L1 C-terminal–deficient splicing variants in patients who were relapsed from PD-L1 blockade therapy. (A)** Representative computed tomographic images of JFCR-119 and JFCR-151 at baseline and at the time of relapse. Bars, 5 cm. **(B)** Representative IHC staining of CD8 and PD-1 at baseline and in the relapsed tumor from JFCR-119. Bars, 10 µm. **(C)** Sashimi plot RNA-seq analysis of the *PD-L1* spliced region. The figure shows representative data for PD-L1v178 (above) and PD-L1v242 (below) in MR203. **(D)** Integrative genomics viewer (IGV) data indicating PD-L1v242 in MR199 and MR203 are shown. **(E)** The PD-L1 splicing variants identified from JFCR-119 and JFCR-151. The domains are indicated as follows: signal peptide (Sig) on 1–18 aa as green; IgV domain on 19–127 aa as yellow; IgC domain on 133–225 aa as pink; transmembrane domain (TM) on 239–259 aa as blue; and cytoplasmic domain (Cyto) on 260–290 aa as gray. The red region demonstrates the additional amino acids from aberrant splicing. **(F)** IGV data of *TARDBP* on the mutated region in pretreatment and relapsed samples of JFCR-119. **(G)** Relapsed tumor-specific mutations in JFCR-119 analyzed by RNA-seq.

To investigate the resistance mechanisms to aPD-L1 blockade, we analyzed two NSCLC patients’ tumors from the lesion which were relapsed to PD-L1 blockade therapy; one patient (JFCR-119) exhibited squamous NSCLC provided a partial response (time to progression [TTP] 9 mo) and another (JFCR-151) diagnosed with lung adenocarcinoma had long stable disease (TTP 14 mo; Fig. 1 A). Through targeted amplicon and whole exon sequencing analyses, we determined that JFCR-119 harbored an active AKT1 mutation (E17K; Figs. S1 A and S5 B) and JFCR-151 carried an epidermal growth factor receptor (EGFR) activating mutation (L858R). To further analyze the resistance mechanisms, we conducted RNA-seq analysis by comparing the pre- and post-treatment bulk tumors from JFCR-119. Neither mutation nor gene expression change in *JAK1*, *JAK2*, *B2M*, *HLA-A*, *HLA-B*, and *HLA-C* was observed (Fig. S1 A) between pre- and post-treatment tumor samples, which were previously indicated to be associated with resistance to PD-1 antibody treatment in melanoma (Zaretsky et al., 2016; Shin et al., 2017). The PD-1 signaling–related gene set was enriched in the relapsed tumor (Fig. S1, B and C). Although we found a few small lesions that lost the expression of HLA and B2M in the relapsed tumor from JFCR-119 (Fig. S1 D), both HLA and B2M were widely positive in the pretreatment tumor and in the majority of post-treatment tumor lesions (Fig. S1, D and E). We also observed that CD8- or PD-1–stained cells had infiltrated into the HLA-positive relapsed specimen (Fig. 1 B and Fig. S1 D). These data suggested the presence of additional resistant mechanisms to PD-L1 blockade therapy.

Further RNA-seq analysis identified a C-terminal–deficient splicing variant of PD-L1 with missing 106 nucleotides in exon 4 (PD-L1v178) and truncated from g724 in exon 5 (PD-L1v242). In addition, PD-L1v242 was found in 2 of 15 patients who acquired resistance to aPD-L1 treatments (Fig. 1, C and D; and Table S1 A). In total, we identified five PD-L1 splicing variants: PD-L1v178 from the JFCR-119 baseline; PD-L1v178, PD-L1v174, PD-L1v242 (same as Fig. 1 D), and PD-L1v265 from the JFCR-119 resistant tumor; and PD-L1v229 from the JFCR-151 relapsed tumor (Fig. S1, G–K; and Fig. S2). All five PD-L1 variants maintained the binding domain to PD-1 (IgV); however, four of them lacked the transmembrane domain (Fig. 1 E). On the basis of data obtained from targeted amplicon sequencing and PCR amplicon sequencing using genomic DNA, there were no relapsed tumor–specific mutations in the splicing junction and intronic region in *PD-L1*, suggesting that these PD-L1 variants resulted from aberrant splicing. Variant specific PCR that was performed successfully validated the specificity of PD-L1v242 in relapsed tumor from JFCR-119 (Fig. S1 F). In addition, we identified mutations in the C-terminal region of TAR DNA binding protein (TARDBP; also known as TDP-43) in relapsed tumor biopsies in JFCR-119 and JFCR-151. Mutations in the C-terminal region of TDP-43 have been reported in patients with amyotrophic lateral sclerosis (ALS), and some of those mutations have been demonstrated to induce aberrant RNA splicing in vivo (Arnold et al., 2013; Fig. 1, F and G; and Fig. S1 L). To test whether mutated TDP-43 interferes PD-L1 splicing, we cloned the genomic sequence of PD-L1 from exon 4 until the end of open reading frame (ORF; PD-L1 ex4–7). By transiently transfecting TDP-43 and PD-L1 ex4–7, we found that the transfected genomic sequence of PD-L1 ex4–7 was successfully spliced in 293FT cells and overexpression of TDP-43 affected the splicing pattern of PD-L1 (Fig. S1 H).

### PD-L1 C-terminal–deficient splicing variants identified from relapsed tumor are highly secreted

**Figure 2.**
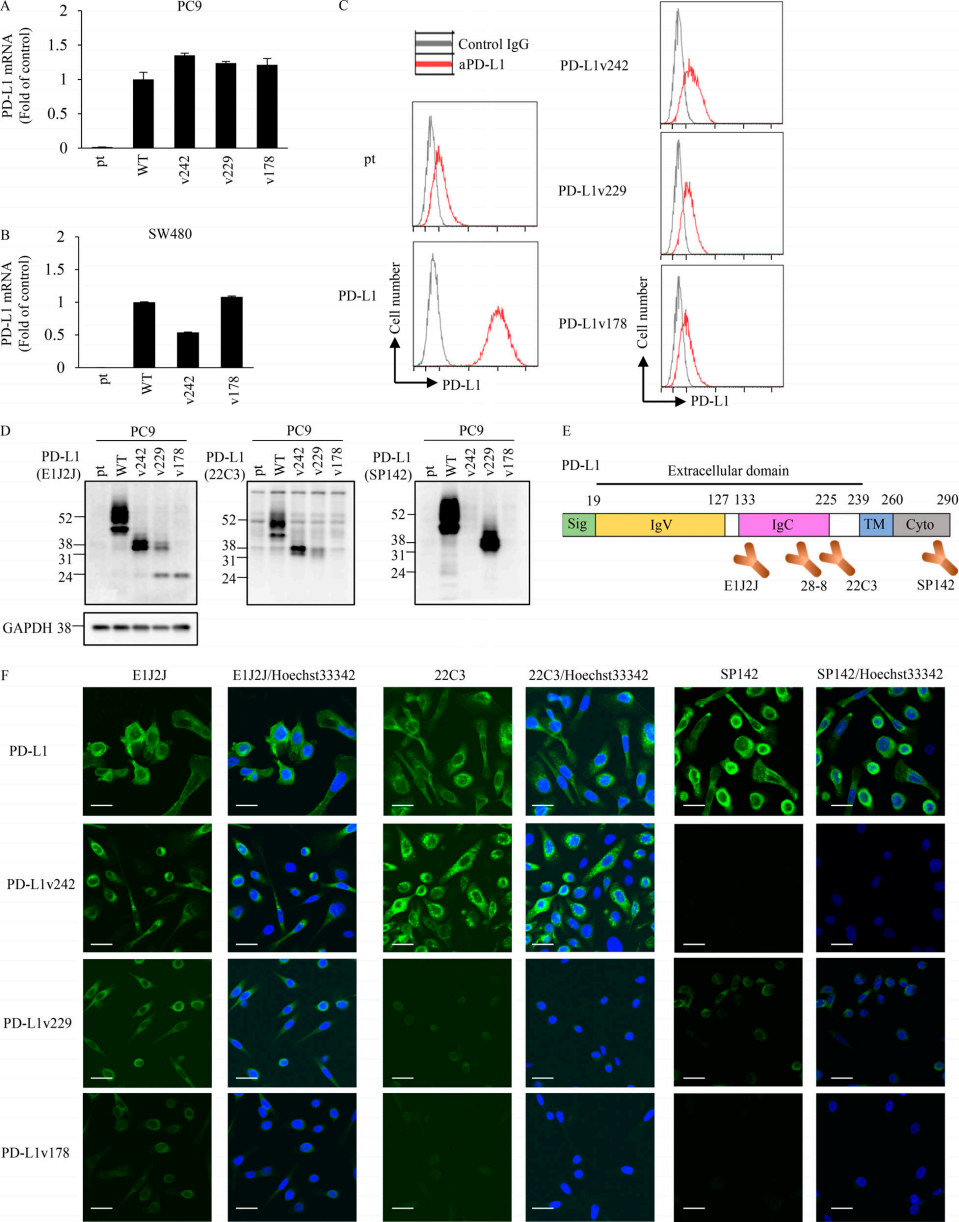
**The recognition pattern of PD-L1 splicing variants by aPD-L1 antibodies. (A and B)** PD-L1 mRNA levels in PD-L1 splicing variant overexpressing PC-9 (A) or SW480 (B) parental (pt) cells were quantified with real-time PCR. The results from three independent experiments are expressed as mean ± SD normalized by that of PC-9/PD-L1 or SW480/PD-L1, respectively. **(C)** Flow cytometric analysis of PD-L1 expression on cell surface in parental, WT, and PD-L1 variants expressing PC-9 cells. **(D and F)** PD-L1-WT and splicing variants with different aPD-L1 antibodies were detected by Western blotting (D) and immunofluorescence staining (F). Bars, 10 µm. **(E)** The epitopes of the aPD-L1 antibodies were roughly estimated by Western blotting and immunofluorescence staining. C and F were independently performed twice, yielding similar results. D was conducted once.

PC-9 and SW480 cell lines with stably overexpressing PD-L1 variants with equivalent PD-L1 mRNA levels were established to further characterize PD-L1 splicing variants (Fig. 2, A and B). Wild-type PD-L1 (PD-L1-WT) was strongly detected on the cell surface, whereas transmembrane domain–deficient PD-L1 variants (PD-L1v242, PD-L1v229, and PD-L1v178) were not detected on the plasma membrane by flow cytometry (Fig. 2 C). Antibodies known to recognize PD-L1 (such as 22C3 and SP142, which are used for companion diagnostics for PD-L1/PD-1 blockade) were tested to confirm their interactions with the identified PD-L1 splicing variants. Western blot analysis showed that clone E1J2J detected all the PD-L1 splicing variants (Fig. 2, D and E; and Fig. S1 O). Clones 28-8 and 22C3 both recognized PD-L1-WT and PD-L1v242, but they did not recognize PD-L1v178. Clone 28-8 seemed to have a greater affinity for PD-L1v229 than 22C3 did (Fig. 2, D and E; and Fig. S1 N). Interestingly, clone SP142, whose epitope is known to be in the C-terminal region of PD-L1, recognized the secreted splicing variant “PD-L1v229”; in PD-L1v229, amino acids coded by exon 7 (part of the intracellular domain) were directly linked to those coded by exon 4 (part of extracellular domain) by in-frame exon 5–6 skipping (Fig. 2, D and E). Results of immunofluorescence staining using the same antibodies were consistent with those of Western blotting, and we further observed that PD-L1-WT tended to localize in the cell membrane and intracellular membrane system, whereas PD-L1v242 and PD-L1v229 localized in cytoplasmic granules (Fig. 2 F).

**Figure 3.**
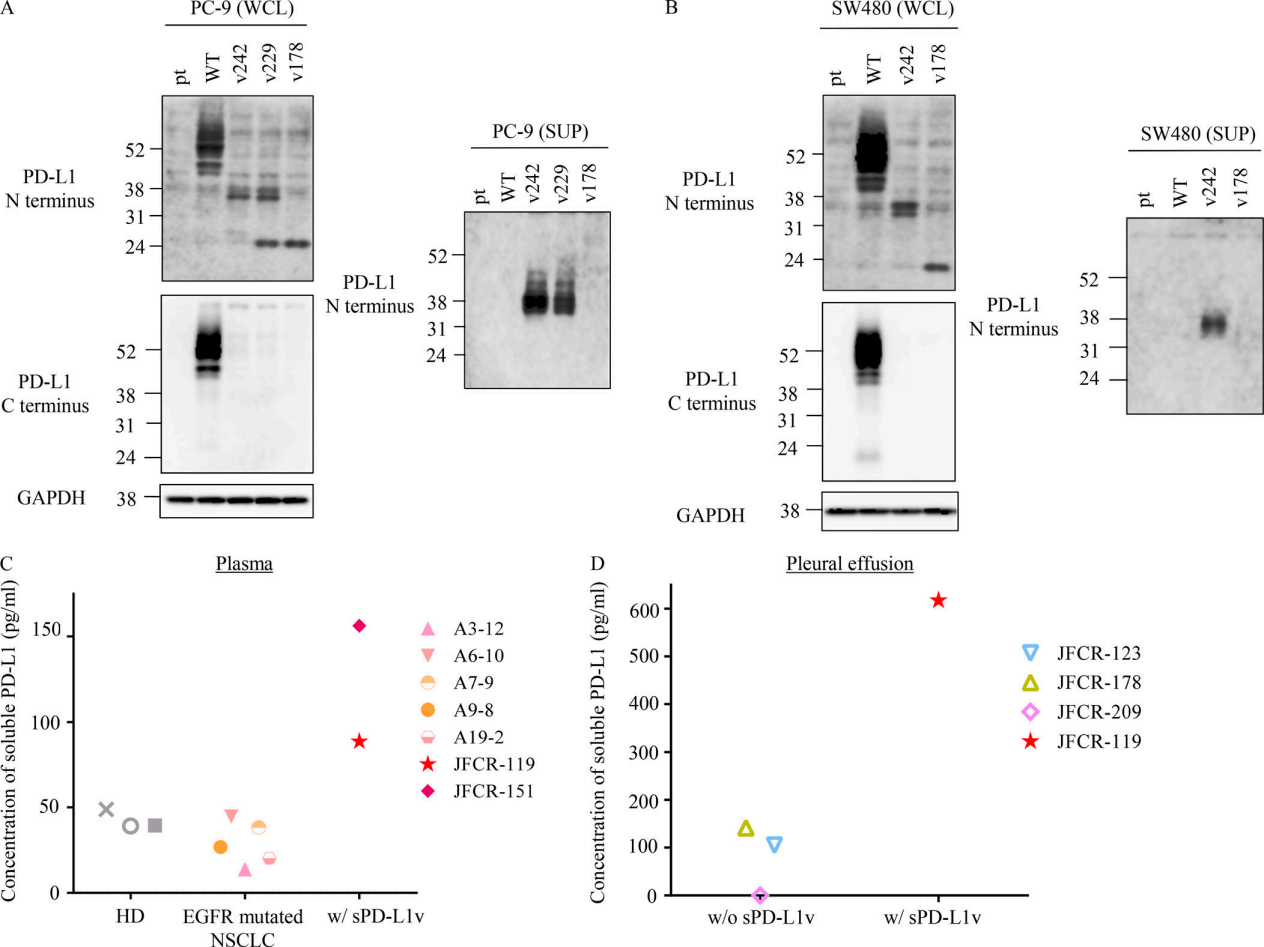
**PD-L1 C-terminal–deficient splicing variants in the relapsed tumor were secreted**. **(A and B)** WCL from PC-9 (A) and SW480 (B) cells which were overexpressed each PD-L1 variants as indicated were analyzed by Western blot. The culture supernatants (SUP) were analyzed following acetone precipitation. **(C and D)** Quantitative analysis of soluble PD-L1 in plasma (C) and pleural effusion (D) from healthy donors (HD), EGFR-mutated NSCLC patients, and patients with the detected PD-L1 splicing variants (JFCR-119 and JFCR-151). Each experiment was independently performed twice, yielding similar results.

Because the PD-L1 C-terminal–deficient splicing variants lost their transmembrane domain, we tested whether they can be secreted. We found that PD-L1v242 and PD-L1v229, which were specifically identified from the relapsed lesions, but not PD-L1-WT, were strongly detected in the culture supernatant (Fig. 3, A and B). To our surprise, PD-L1v178, which was reported to be secreted in a previous study (Zhou et al., 2017), was barely detected in our current study (Fig. 3, A and B), suggesting at least that PD-L1v242 and PD-L1v229, specifically identified from the relapsed tumor, were highly secreted. To confirm these observations in patients with relapsed tumors, we evaluated the level of soluble PD-L1 in plasma and pleural effusion near the lesions. It has been reported that the exosome fraction in plasma carries PD-L1 on its surface (Chen et al., 2018); therefore, we tested exosome-free plasma after ultracentrifugation at 100,000 *g* for 90 min. The plasma concentrations of soluble PD-L1 were higher in patients with sPD-L1 variants than in healthy donors and patients with EGFR-mutated NSCLC (Fig. 3 C). In addition, the soluble PD-L1 level in pleural effusion fluid from JFCR-119 (taken from near the tumor lesions) was sixfold higher than that in patients without sPD-L1 variants and that in the patient’s own plasma (Fig. 3, C and D). This suggested that soluble PD-L1 from a tumor might accumulate locally.

**Figure 4.**
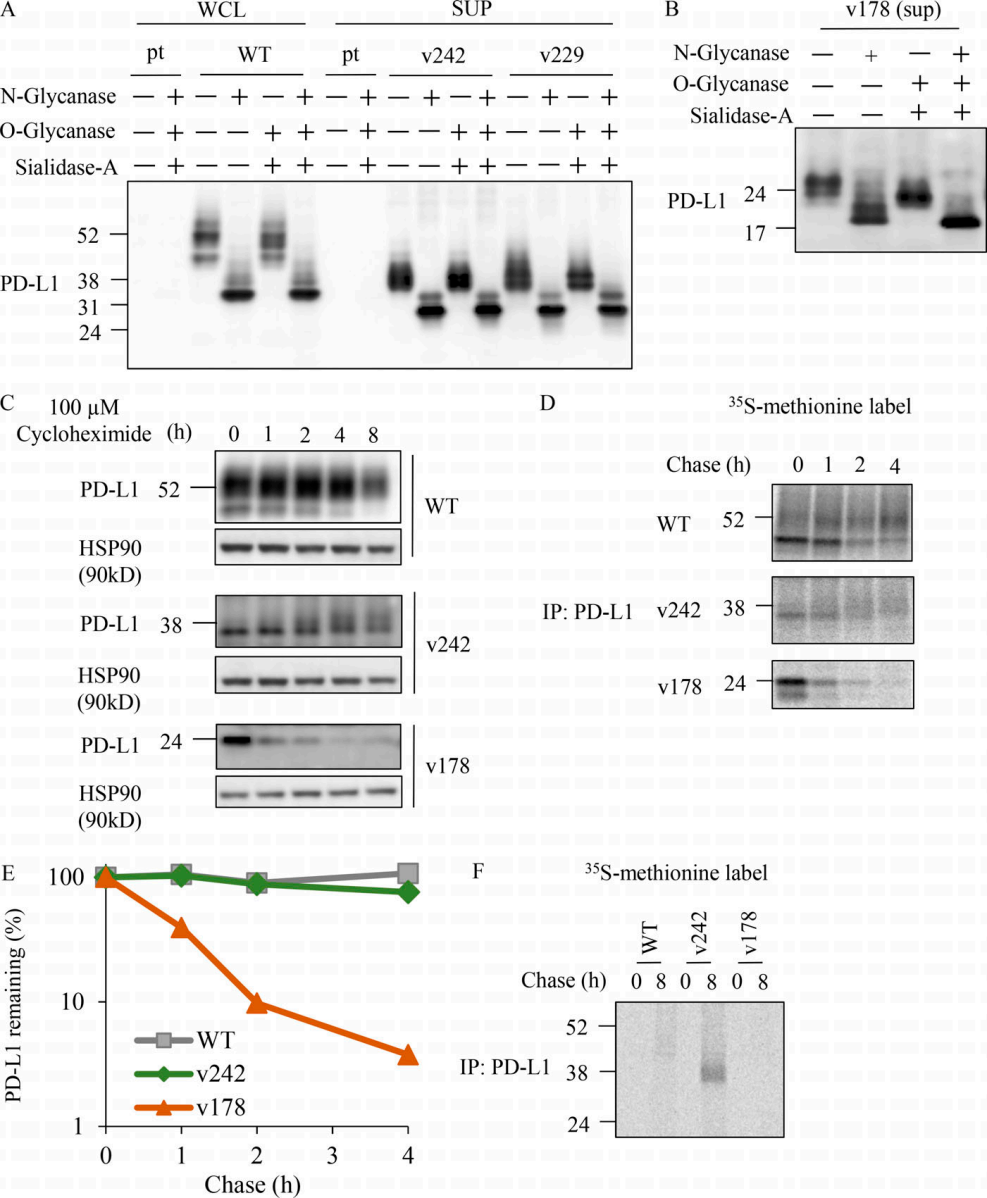
**PD-L1 C-terminal–deficient splicing variants from relapsed tumor were stable. (A and B)** Glycosylation analysis of sPD-L1 variants. WCL of PC-9 parental cell (pt) and PC-9/PD-L1, or concentrated sPD-L1 variants obtained from culture supernatant (SUP) of PC-9/PD-L1v242, PC-9/PD-L1v229 (A), and PC-9/PD-L1v178 (B) were treated with N-glycanase, sialidase-A, or O-glycanase for 3 h at 37°C and then analyzed by Western blot. **(C)** Immunoprecipitated samples from WCL and culture supernatant with aPD-L1 antibody were analyzed by Western blot. HSP90 in WCL were used as the loading control. **(D)** ^35^S-labeled methionine cells were cultured in a radio-free medium for the indicated period. Immunoprecipitated (IP) samples from WCL and the culture supernatant were evaluated with SDS-PAGE and visualized with a phosphor imaging scanner. **(E)** The remaining PD-L1 was quantified with ImageJ software based on the results of (D). **(F)** Phosphor imaging of culture supernatant samples immunoprecipitated by aPD-L1 antibody. C–F were independently performed twice, yielding similar results. A and B were conducted once.

PD-L1 has been reported to harbor four N-glycosylation sites in the extracellular region: N35, N192, N200, and N219. Three of these, N192, N200, and N219, have been indicated to contribute to the stabilization of PD-L1 and were maintained in PD-L1v242 and PD-L1v229 (Li et al., 2016). To further investigate the characteristics of the PD-L1 splicing variants, we analyzed the glycosylation status of PD-L1v242, PD-L1v229, and PD-L1v178. We observed that the molecular weight was reduced following treatment with N-glycanase but not with O-glycanase and sialidase A, indicating these PD-L1 splicing variants were N-glycosylated (Fig. 4, A and C). In addition, PD-L1v178 exhibited a minimal band shift compared with PD-L1-WT, PD-L1v242, and PD-L1v229 (Fig. 4 B), possibly because of the smaller number of N-glycosylation sites (lacking N192, N200, and N219). Fc-conjugated podoplanin, a factor that promotes platelet aggregation, was used as the positive control to confirm the enzyme activity of O-glycanase and N-glycanase (Fujita and Takagi, 2012; Takemoto et al., 2017; Fig. S1 P). We further confirmed that PD-L1v242, but not PD-L1v178, was stable and secreted in the protein half-life assay with cycloheximide and pulse-chase assay using ^35^S-labeled methionine, supporting that the N-glycosylation status on PD-L1v242 and PD-L1v229 might contribute to its stable secretion (Fig. 4, C–F).

### sPD-L1 splicing variants bind to PD-1

**Figure 5.**
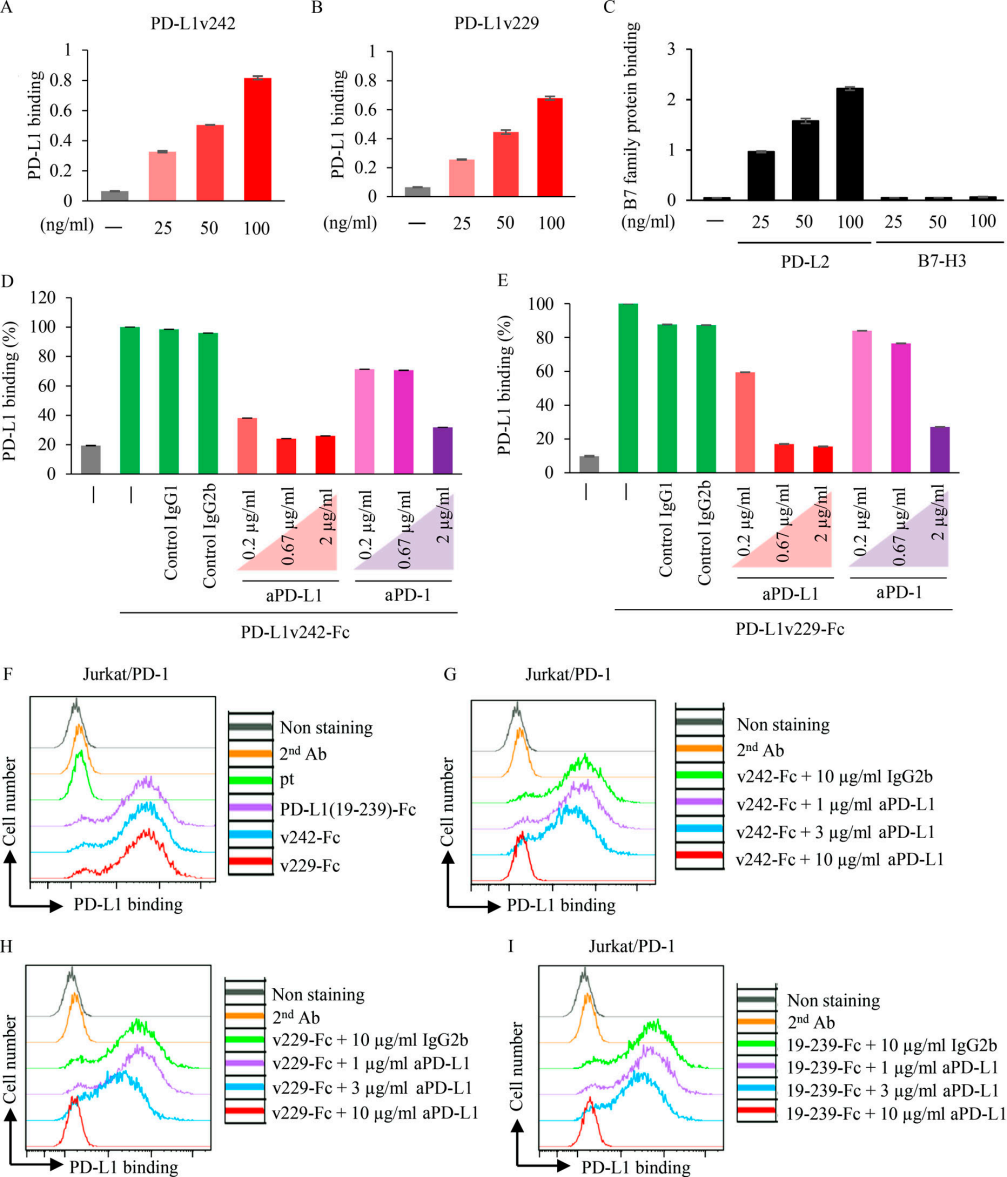
**sPD-L1 splicing variants bind to PD-1. (A–C)** The binding of PD-L1v242 (A), PD-L1v229 (B), PD-L2, and B7-H3 to PD-1 (C) were evaluated by ELISA (*n* = 3). Results are expressed as mean ± SD. **(D and E)** Purified Fc-tagged PD-L1v242 (D) and PD-L1v229 (E) preincubated with or without aPD-L1 or aPD-1 antibody for 30 min was incubated in PD-1 coated wells for 2 h at RT. The binding of PD-L1 variants to PD-1 was detected based on the absorbance at 450 nm (*n* = 3). Results are expressed as mean ± SD. **(F–I)** Flow cytometry analysis for evaluating sPD-L1 splicing variants binding to PD-1. Culture supernatant from CHO parental cell (pt) and those overexpressing PD-L1v242-Fc (F and G), PD-L1v229-Fc (F and H), and PD-L1(19–239)-Fc (F and I) was incubated with Jurkat/PD-1 cells in the condition described for 1 h. Fc-tagged PD-L1 variant binding to PD-1 was evaluated with flow cytometer. Each experiment was independently performed twice, yielding similar results. The data of nonstaining and secondary antibody (2nd Ab) as negative control in F–I were the same.

To evaluate the function of PD-L1 splicing variants, we focused on the stable sPD-L1 splicing variants (PD-L1v242 and PD-L1v229) identified from the relapsed lesions. An ELISA system coated with His-tagged PD-1 was used to investigate the interactions between the sPD-L1 splicing variants and PD-1. We observed that PD-L1v242 and PD-L1v229 were bound to PD-1 in a dose-dependent manner (Fig. 5, A and B). As a control, we confirmed that ELISA can detect the binding of PD-L2, but not B7-H3, to PD-1 (Fig. 5 C). In addition, the binding was interrupted by the aPD-L1 and aPD-1 antibodies (Fig. 5, D and E). Consistent with these results, the binding between PD-L1 variants and PD-1 was also detected in a dose-dependent manner using flow cytometry (Fig. 5, F–I), indicating that sPD-L1 splicing variants could bind to PD-1.

### sPD-L1 splicing variants disturb PD-L1 blockade by trapping aPD-L1 antibody

**Figure 6.**
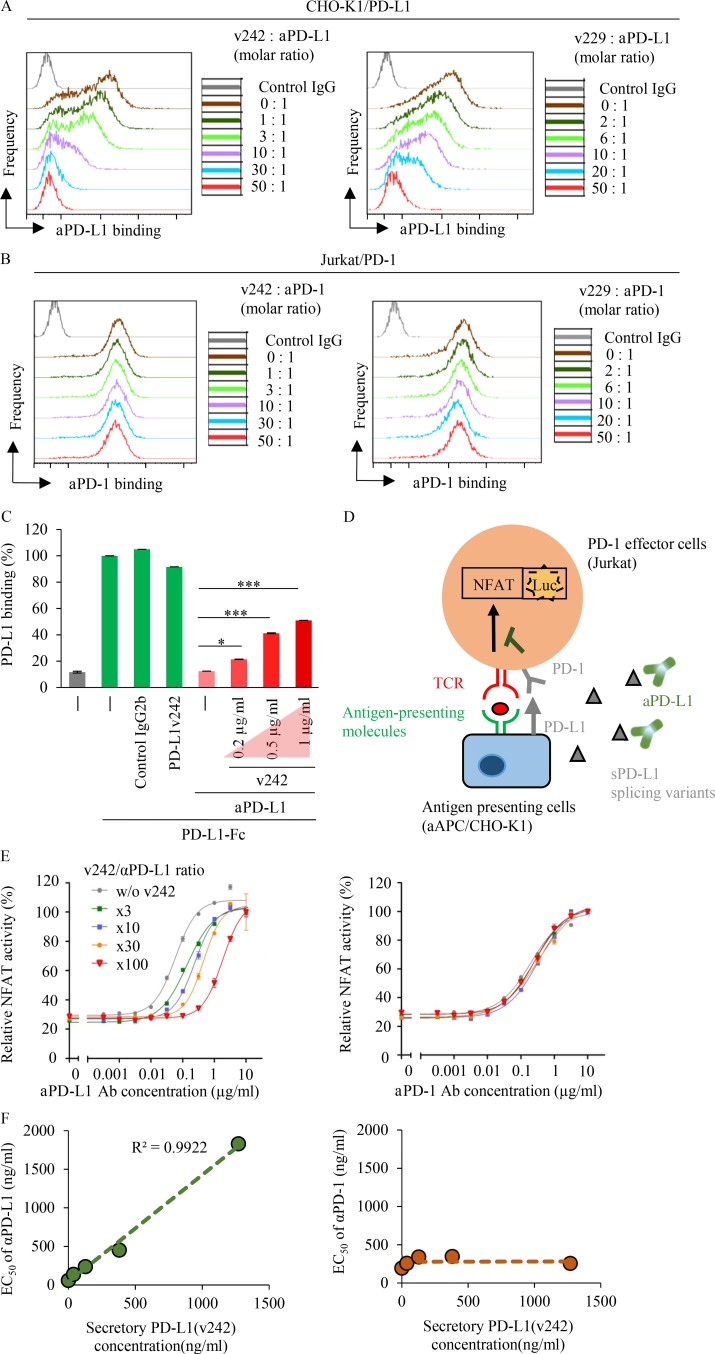
**sPD-L1 splicing variants attenuate the neutralizing activity of aPD-L1 by trapping the antibody. (A and B)** Flow cytometric analysis of aPD-L1 antibody binding to PD-L1 (A) and aPD-1 antibody binding to PD-1 (B) in the presence of indicated sPD-L1 variants molar ratio. **(C)** Fc-tagged PD-L1 and aPD-L1 antibodies (0.75 µg/ml) preincubated with or without PD-L1v242 as indicated were added to PD-1 precoated 96-well ELISA plates. The binding of Fc-tagged PD-L1 to PD-1 was detected based on absorbance at 450 nm; 0.2 µg/ml PD-L1v242 is equal to 0.75 µg/ml aPD-L1 antibody in molar ratio. *n* = 3. Results are expressed as mean ± SD. Paired two-tailed Student *t* test was used. *, P < 0.05; ***, P < 0.001. **(D)** Schematic diagram illustrating the NFAT-luc assay for evaluating TCR-mediated NFAT transduction. **(E)** The neutralizing activity (EC_50_) of aPD-L1 and aPD-1 antibodies was determined using NFAT-luc assay in the presence of various molar ratios of PD-L1v242 to the antibody (*n* = 3). Results are expressed as mean ± SD. **(F)** The correlation between PD-L1v242 and the EC_50_ of the antibody according to the Lineweaver–Burk plot was analyzed based on the results of (E), which suggested that the sPD-L1 splicing variants reduce the aPD-L1 inhibitory activity competitively. Each experiment was independently performed twice, yielding similar results.

Secreted variant proteins often work as “decoys” contributing to drug resistance. For instance, secreted CD20 has been indicated to be one of the resistance mechanisms against rituximab, an anti-CD20 antibody (Smith, 2003). This led us to hypothesize that PD-L1v242 and PD-L1v229 act as decoys to PD-L1 blockade and attenuate its neutralizing activities. We used a commercially available blockade antibody of the PD-L1/PD-1 axis to evaluate the antibody binding to its target protein in the presence of purified PD-L1v242 and PD-L1v229 through flow cytometry and ELISA analysis. Our results indicated that both PD-L1v242 and PD-L1v229 attenuated aPD-L1 antibody binding to PD-L1 dose dependently (Fig. 6, A–C), thus supporting the idea that sPD-L1 splicing variants can work as decoys to PD-L1 blockade.

### sPD-L1 splicing variants interrupt the reactivation of NFAT-regulated signal transduction by PD-L1 blockade

The PD-L1/PD-1 axis has been reported to negatively regulate T cell activation by inhibiting TCR-mediated signal transduction including NFAT-regulated signal transduction (Rao et al., 1997; Crabtree and Olson, 2002). To evaluate whether NFAT activity is negatively regulated by PD-L1/PD-1 interaction, we used CHO-K1 cells that expressed antigen-presenting molecules to activate cognate TCRs as antigen-presenting cells (aAPC/CHO-K1), and PD-1 expressing Jurkat T cells harboring matched TCR and luciferase reporters driven by an NFAT-response element (PD-1 effector cells). The NFAT activity increased when the PD-1 effector cells were cocultured with the aAPC/CHO-K1 cells (Fig. S3 A). In contrast, PD-L1 overexpression (PD-L1 aAPC/CHO-K1) suppressed the NFAT activity of PD-1 effector cells, whereas the activity was reactivated by aPD-L1 or aPD-1 antibody dose dependently (Fig. S3 A).

To further investigate the function of the sPD-L1 splicing variants in PD-L1 blockade, we evaluated the NFAT activity in the presence of the sPD-L1 variants (Fig. 6 D). Although sPD-L1 splicing variants had no direct effect on suppression of the NFAT activity in Jurkat cells (Fig. S3 B), NFAT activation by the aPD-L1 antibody can be interrupted by adding threefold higher concentration of aPD-L1 variants (Fig. S3 C). Conversely, the activation of NFAT by the aPD-1 antibody was not affected by sPD-L1 variants (Fig. 6, E and F; and Fig. S3, D and E). In testing whether the secreted protein traps its antibody as a decoy in general, we found that soluble PD-1 also has the potential to influence EC_50_ of aPD-1 antibody (Fig. S3, F, G, J, and K). Based on these results, we identified a linear relationship between the dose of the sPD-L1 variants and the EC_50_ of the aPD-L1 antibody, which suggested that sPD-L1 splicing variants are able to trap aPD-L1 antibodies in a competitive manner to prevent T cell reactivation in the tumor environment (Fig. 6 F and Fig. S3, H and I).

### sPD-L1 splicing variants contribute the resistance to PD-L1 blockade treatment

**Figure 7.**
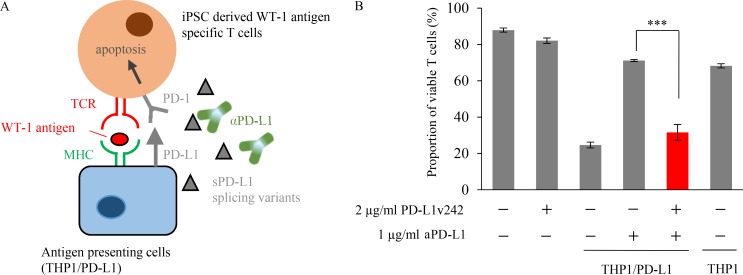
**sPD-L1 splicing variants contribute the resistance to PD-L1 blockade in WT-1 tumor antigen–specific iPSC-derived CD8 T cell model. (A)** Schematic illustration of apoptosis assay for testing whether PD-L1v242 attenuates the blockade effect of aPD-L1 antibody. **(B)** iPSC-derived WT-1–specific T cells overexpressing PD-1 were cocultured with THP-1 cells overexpressing PD-L1 for 18 h in the presence of aPD-L1 antibody (1 µg/ml) or PD-L1v242 (2 µg/ml). The dead cell ratio was flow cytometrically measured using propidium iodide staining, and bars represent the proportion of live T cells in comparison with those before coculture. 2 µg/ml of PD-L1v242 was approximately eight times more than 1 µg/ml aPD-L1 antibody in molar ratio. The results are representative from three independent experiments and are shown as mean ± SD (*n* = 3). Paired two-tailed Student *t* test was used. ***, P < 0.001. The experiment was independently performed twice, yielding similar results.

**Figure 8.**
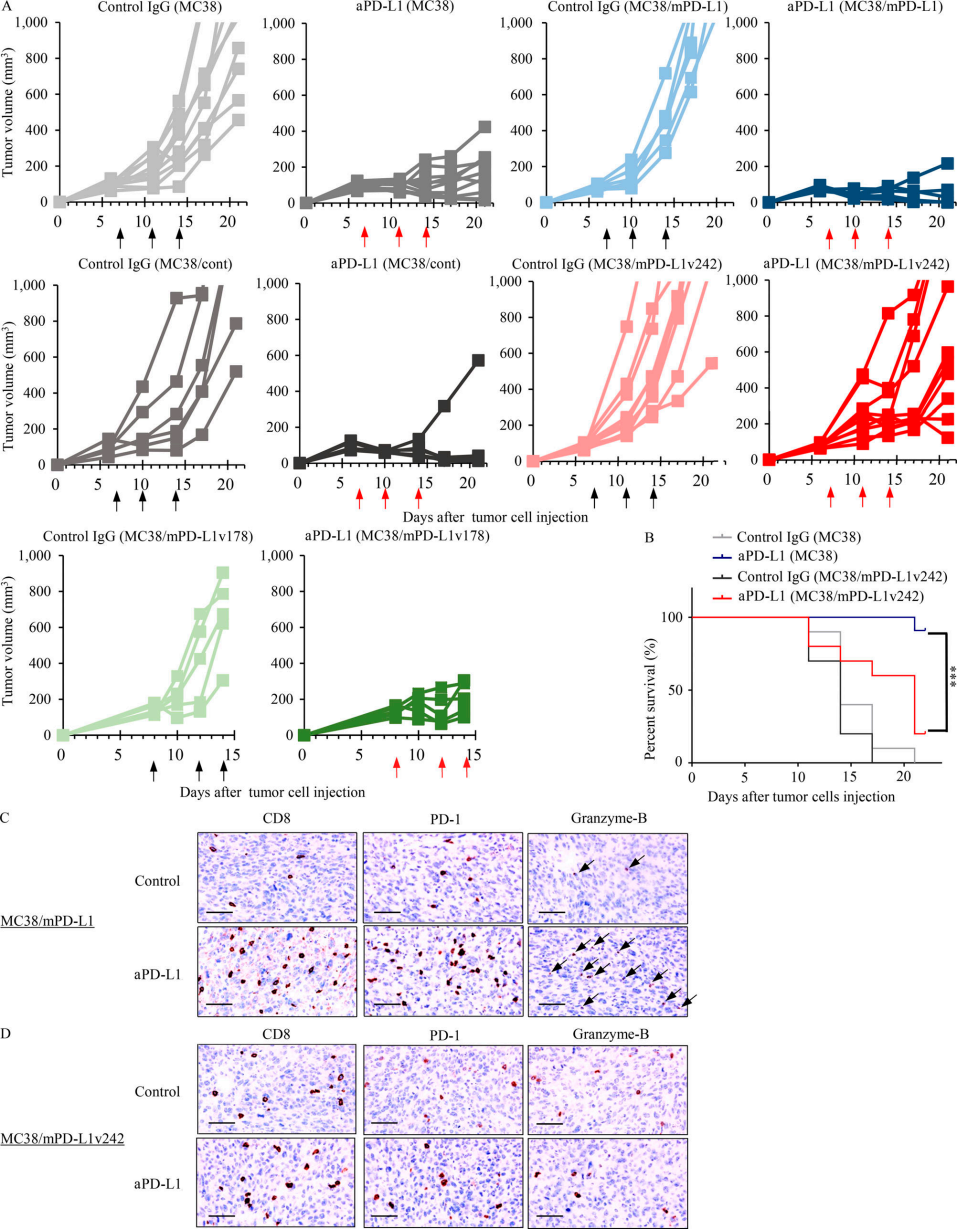
**sPD-L1 splicing variants mediate the resistance to PD-L1 blockade in MC38 syngeneic mouse model. (A)** C57BL/6 mice bearing MC38/cont (*n* = 6), MC38/mPD-L1 (*n* = 6), MC38 (*n* = 10), MC38/mPD-L1v242 (*n* = 10), and MC38/mPD-L1v178 (*n* = 5) were intraperitoneally administrated 50 µg/mouse control IgG or 35 µg/mouse aPD-L1 antibody. The schedules for treatment are indicated by black arrows (control IgG) and red arrows (aPD-L1). Tumor volume is plotted individually. **(B)** Kaplan–Meier survival curves for mice bearing MC38 or MC38/mPD-L1v242. The survival curves were compared by applying the Gehan–Breslow–Wilcoxon test. ***, P < 0.001. **(C and D)** Representative IHC staining of mouse CD8, PD-1, and granzyme B was performed on day 21 for MC38/mPD-L1 (C) and MC38/mPD-L1v242 (D) xenograft tumors. Bars, 20 µm. Each experiment was independently performed twice, yielding similar results.

We previously established induced pluripotent stem cell (iPSC)–derived WT-1 tumor antigen–specific T cells to experimentally evaluate the efficacy of adoptive transfer therapy (Maeda et al., 2016). To further investigate whether these cytotoxic T cells are negatively regulated by the PD-L1/PD-1 pathway, we established PD-1–overexpressing regenerated WT-1 antigen-specific iPS-T cells (iPS-reT/PD-1). We successfully observed that iPS-reT/PD-1 cells exhibited significant reduction of cell viability following coculturing with PD-L1 overexpressing WT-1 antigen-presenting THP-1 cells (Fig. 7 A), which might mimic intratumoral CD8^+^ T cell apoptosis induced by PD-L1/PD-1 interaction (Horton et al., 2018). To further test the effect of sPD-L1 splicing variants on human T cells, we examined the viability of iPS-reT/PD-1 cells by adding both aPD-L1 antibody and sPD-L1 variants. Treatment of aPD-L1 antibody significantly recovered the cell viability of iPS-reT/PD-1 cells in the presence of WT-1 antigen-presenting THP-1 cells, whereas the coculture of spliced sPD-L1 variants significantly reduced the survival of iPS-reT/PD-1 cells by interrupting the neutralizing activity of aPD-L1 antibody (Fig. 7 B). To extend these observations to an in vivo setting, we produced mouse PD-L1 variants (mPD-L1v242 and mPD-L1v178), which have the same human PD-L1 truncation by aberrant splicing (Fig. S4), and overexpressed them in MC38, a cell line from C57BL/6 murine colon adenocarcinoma cells which has been reported to have high PD-L1 expression induced by IFN-γ and a response to the blockade of the PD-1/PD-L1 axis (Juneja et al., 2017). Through the administration of aPD-L1 antibody to mice bearing MC38, MC38/cont, MC38 overexpressing mouse PD-L1 (MC38/mPD-L1), MC38/mPD-L1v242, and MC38/mPD-L1v178 (Fig. 8 A), we confirmed that MC38/mPD-L1v242 was resistant to aPD-L1 antibody treatment and the median survival was shorter than that of MC38 (Fig. 8 B). Interestingly, PD-L1v178, which was barely secreted, did not promote aPD-L1 treatment resistance (Fig. 8 A). Further immunohistochemistry (IHC) analysis of the tumor environment showed that the number of CD8- and PD-1–positive cells increased significantly in the MC38/mPD-L1 tumor lesion after the PD-L1 blockade treatment; this increase was not observed in MC38/mPD-L1v242. Consistent with these results, granzyme B–positive cells were widely located in MC38/mPD-L1 but not in MC38/mPD-L1v242 after aPD-L1 treatment; this suggested that the PD-L1 blockade lost its activity of reactivating cytotoxic lymphocyte by PD-L1v242 (Fig. 8 D).

**Figure 9.**
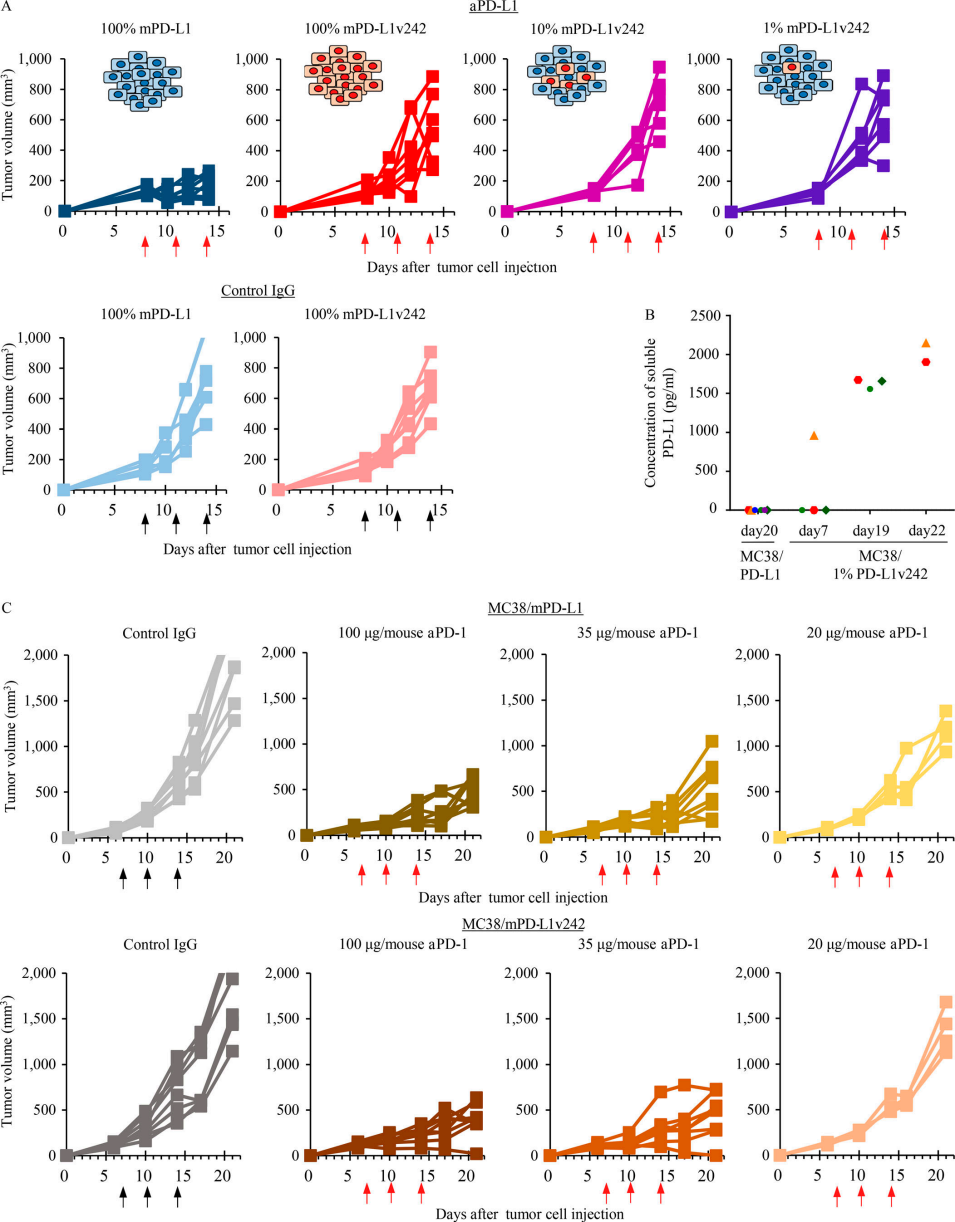
**aPD-1 treatment overcame the resistance to PD-L1 blockade induced by sPD-L1 splicing variants in MC38 syngeneic mouse model. (A)** C57BL/6 mice bearing MC38/mPD-L1 (*n* = 7), MC38/mPD-L1v242 (*n* = 7), 10% MC38/mPD-L1v242 + 90% MC38/mPD-L1 (*n* = 7), and 1% MC38/mPD-L1v242 + 99% MC38/mPD-L1 (*n* = 7) were intraperitoneally administered 35 µg/mouse control IgG or 35 µg/mouse aPD-L1 antibody. The schedules for treatment are indicated by black arrows for control IgG and red arrows for aPD-L1. The plot shows the tumor volumes for each mouse. **(B)** The plasma levels of soluble PD-L1 in mice bearing MC38/mPD-L1 and 1% MC38/mPD-L1v242 were sequentially evaluated by ELISA. To remove the exosome fraction, the plasma was ultracentrifuged at 100,000 *g* for 90 min. **(C)** C57BL/6 mice bearing MC38/mPD-L1 and MC38/mPD-L1v242 were intraperitoneally administrated either 100 µg/mouse control IgG (*n* = 8) or aPD-1 antibody at doses of 100 µg/mouse (*n* = 8), 35 µg/mouse (*n* = 8), or 20 µg/mouse (*n* = 4). The treatment days are indicated by black arrows for control IgG and red arrows for aPD-1. The plot shows the tumor volumes for each mouse. A was independently performed twice, yielding similar results. B and C were conducted once.

**Figure 10.**
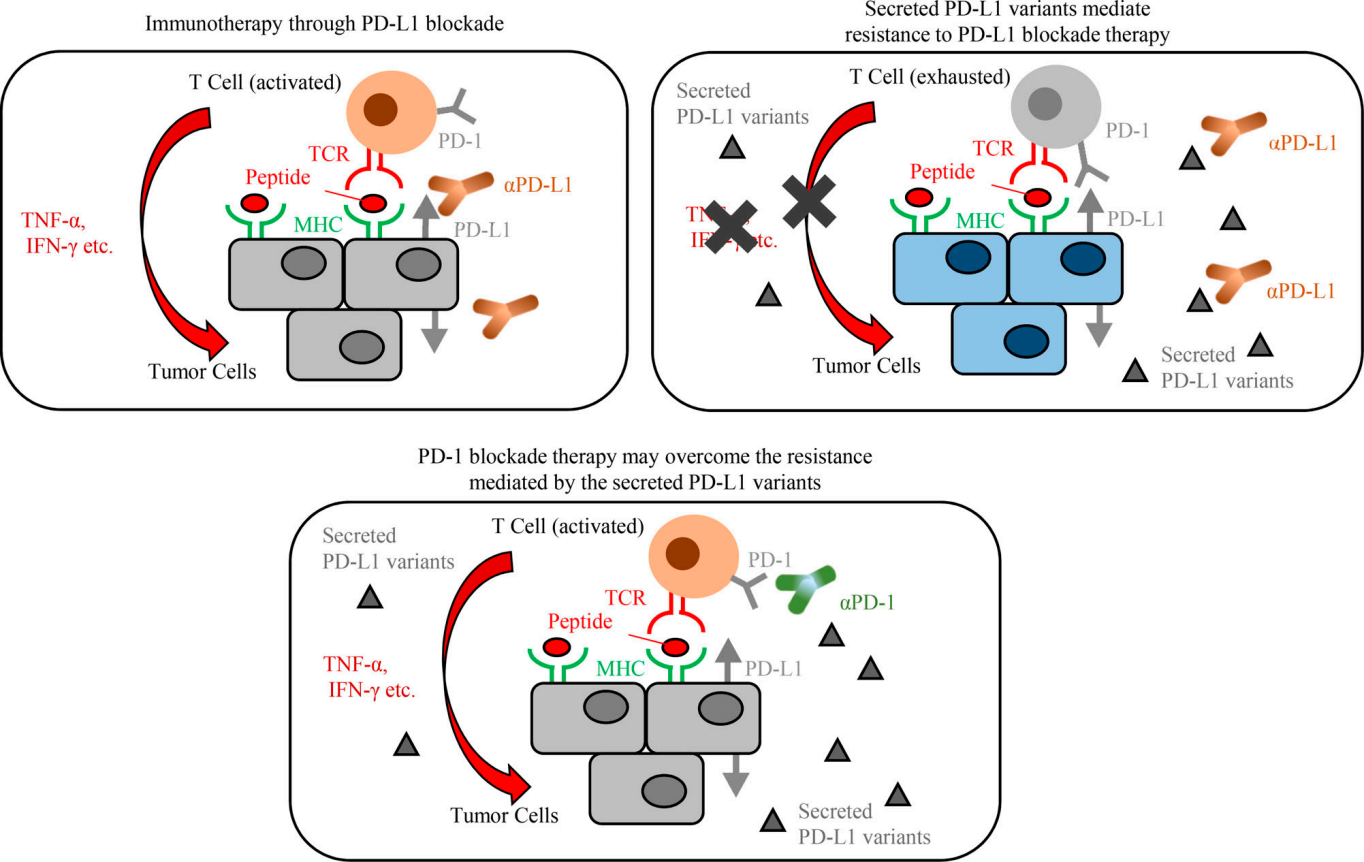
**The proposed model for the sPD-L1 splicing variants associated with resistance to aPD-L1 antibody treatment.**

To further investigate whether a small fraction of cancer cells that harbor sPD-L1 variants would protect the whole tumor from attack by cytotoxic lymphocytes, we tested the PD-L1 blockade antibody in mice subcutaneously injected with a mix of MC38/PD-L1v242 and MC38/PD-L1-WT in various ratios. Unexpectedly, a mixture of only 1% of cells expressing PD-L1v242 and 99% of cells expressing PD-L1-WT successfully induced PD-L1 blockade resistance (Fig. 9 A), even though the level of soluble PD-L1 in plasma was low on day 7 (the first day of treatment). The concentration of soluble PD-L1 eventually increased in mice bearing 1% MC38/PD-L1v242, although the plasma-soluble PD-L1 level was undetectable in mice bearing wild-type PD-L1 overexpressed MC38 (MC38/PD-L1-WT), even when their tumor volume exceeded 1,000 mm^3^ (Fig. 9 B). This suggested that PD-L1 variants secreted in the tumor environment and gradually accumulated in plasma as the tumor progressed. Furthermore, the overexpressed PD-L1v242 in MC38 did not affect the efficacy of aPD-1 treatment in vivo (Fig. 9 C). These results indicate that the sPD-L1 splicing variant works as a decoy of aPD-L1 antibody, contributing to PD-L1 blockade treatment resistance, and aPD-1 therapy may be an option to overcome the resistance (Fig. 10).

## Discussion

Immune checkpoint inhibitors against the PD-L1/PD-1 axis produce clinical benefit. Anti–PD-1 antibody (approved in 2014) and PD-L1 blockades, atezolizumab in urothelial carcinoma and NSCLC, durvalumab in urothelial carcinoma, and avelumab in Merkel cell carcinoma and urothelial carcinoma, were recently approved by the US Food and Drug Administration. However, the incidence of acquired resistance to PD-1 and PD-L1 blockade antibodies is increasing. Several groups have reported resistance to PD-1 blockade, but the mechanisms surrounding resistance to aPD-L1 treatment remain poorly understood.

In the present study, we identified five PD-L1 splicing variants and demonstrated that PD-L1v242 and PD-L1v229 (found in tumors relapsed from aPD-L1 treatment) were able to be secreted stably and induce therapeutic resistance. Although PD-L1v178, which was found in both pretreated and relapsed tumors in JFCR-119, was previously reported in several melanoma cell lines and suggested to be the origin of the circulating PD-L1 in serum (Zhou et al., 2017), in our investigation, PD-L1v178 was unstable and barely secreted and did not associate with the resistance to PD-L1 blockade therapy even though it lacked the transmembrane region. The instability and lack of secretion on PD-L1v178 was possibly caused by lacking N-glycosylation sites; N192, N200, and N219, which are located in the extracellular region, were shown to be critical in stabilizing PD-L1 from degradation (Li et al., 2016).

Secreted proteins have been demonstrated to have several functions. Notch decoy was shown to inhibit tumor growth by distributing angiogenesis (Kangsamaksin et al., 2015). Furthermore, secreted CD20 was reported to be a mechanism of resistance to rituximab, an anti-CD20 antibody (Smith, 2003). In our study, we found that sPD-L1 splicing variants, PD-L1v242 and PD-L1v229, could trap the aPD-L1 antibody and inhibit antibody binding to PD-L1. In addition, PD-L1v242 and PD-L1v229 interrupted the reactivation of NFAT-regulated signal transduction by PD-L1 blockade.

The immunosuppressive function of soluble PD-L1 has been implied in a previous study, in which the correlation between the level of circulating serum PD-L1 and poor prognosis was observed in multiple cancers (Zhu and Lang, 2017). Moreover, circulating serum PD-L1 was suggested to be a biomarker for predicting immune checkpoint inhibitor response, particularly to the blockade of PD-1 and CTLA-4 in malignant melanoma and multiple myeloma (Wang et al., 2015; Zhou et al., 2017). Unfortunately, we could not observe sPD-L1 splicing variants suppressing TCR-mediated NFAT activation, although anti-CD3–stimulated T cell proliferation was previously suppressed using a coated plate with PD-L1 variants (Zhou et al., 2017). PD-1 suppresses T cell activation by transiently forming microclusters with TCRs and the costimulatory receptor CD28 (Hui et al., 2017). Therefore, PD-1 localization might be important when interacting with PD-L1, and soluble spliced PD-L1 might alter the tumor environment for immunosuppression.

We identified mutations in the splicing regulatory factor TDP-43 in relapsed tumors treated with PD-L1 blockade antibodies. Although TDP-43 has been reported to bind to >6,000 pre-mRNAs and to affect the splicing patterns of ∼600 mRNAs (Polymenidou et al., 2011), the current study did not show any clear splicing alterations of the genes in antigen presentation or in the JAK/STAT pathway. To date, mutations in C-terminal region of TDP-43 have been identified in patients with ALS, and several studies have demonstrated that the TDP-43 mutations Q331K and M337V caused ALS in vivo by producing aberrant RNA splicing. In our study, we found two mutations of TDP-43 (W334L in JFCR-119 and R361T in JFCR-151) that were previously observed in patients with ALS. No report has shown a direct impact of the TDP-43 variants on the splicing of PD-L1, but it has been observed that depletion of TDP-43 in the adult mouse brain resulted in up-regulation of PD-L1, which implies some relationship between TDP-43 and PD-L1 (Polymenidou et al., 2011). To evaluate relevance between TDP-43 and PD-L1, we transiently coexpressed TDP-43 and the genomic sequence of PD-L1 from exon 4 to the end of ORF (PD-L1 ex4–7) in 293FT cells. This resulted in the induction of exon 6–skipped splicing of PD-L1, suggesting that TDP-43 can affect the splicing pattern of PD-L1. However, further studies are needed to elucidate the mechanism for the induction of aberrant PD-L1 splicing variants.

Companion diagnostic antibodies for detecting PD-L1 in tumors have been approved in the clinical setting; examples include clone 22C3 for pembrolizumab and clone SP142 for atezolizumab. However, the staining pattern for PD-L1 has been controversial (Fujimoto et al., 2018). In the present study, we evaluated whether these diagnostic aPD-L1 antibodies recognized our spliced PD-L1 variants. We found E1J2J recognized all the PD-L1 variants, and clone 28-8 detected PD-L1v229 more efficiently than 22C3 did. Unexpectedly, clone SP142, which recognizes the intracellular domain of PD-L1, was able to detect secreted spliced variants PD-L1v229. These results suggest that the staining pattern by diagnostic aPD-L1 antibodies may be influenced by the presence of the PD-L1 variants.

In conclusion, we completely identified two sPD-L1 C-terminal splicing variants, PD-L1v242 and PD-L1v229, from four patients who relapsed from PD-L1 blockade therapy. In vitro, these sPD-L1 variants functioned as decoys to PD-L1 blockade antibodies. Furthermore, a mixture of only 1% MC38 cells with sPD-L1 variants (MC38/mPD-L1v242) and 99% of cells that overexpressed wild-type PD-L1 (MC38/mPD-L1) were found to cause resistance to PD-L1 blockade therapy by accumulation of soluble PD-L1 in the tumor and plasma. This finding provides additional understanding of the mechanism and characterization of PD-L1 splicing variants in the aPD-L1 blockade therapy resistance in vitro and in vivo. Consistent with these results, the levels of soluble PD-L1 in plasma and pleural effusion from patients who harbored sPD-L1 splicing variants were significantly higher than those in patients without the variants. We also demonstrated in vivo that aPD-1 antibody treatment overcame the resistance induced by the sPD-L1 variants. Taken together, these findings suggest that the presence of sPD-L1 splicing variants or the level of soluble PD-L1 in plasma or pleural effusion may work as a biomarker to predict a patient’s response to PD-L1 blockade therapy and that aPD-1 antibody treatment could be a therapeutic option to overcome sPD-L1 variant-induced resistance.

## Materials and methods

### Tumor samples

Biopsy samples were obtained from patients enrolled in the clinical trial who were administered PD-L1 blockade antibody.

All patients provided informed consent for genetic and cell biological analyses, which were performed in accordance with protocols approved by the Institutional Review Board of the Japanese Foundation for Cancer Research, the Ethic Committee (Comité de Protection des Personnes Ile-de-France 3), or the Competent Authority (Agence nationale de sécurité du médicament et des produits de santé).

### Next generation sequencing

Genomic DNA and total RNA were extracted with a DNeasy Blood and Tissue Kit and RNeasy Mini Kit (Qiagen), respectively.

Transcriptome libraries are prepared using the TruSeq RNA Library Prep Kit v2 or the TruSeq Stranded mRNA kit (Illumina) following manufacturer’s instruction. Briefly, the kit converts the poly-A containing mRNA from total RNA (1,000 ng engaged in the process) into a cDNA library using poly-T oligo-attached magnetic bead selection. Following mRNA purification, the RNA is chemically fragmented before reverse transcription and cDNA generation. The fragmentation step results in a RNA-seq library that includes inserts that range in size from 300∼400 mers. The cDNA fragments then go through an end repair process, the addition of a single ‘A’ base to the 3′ end and then ligation of the adapters. Finally, the products are purified and enriched with PCR to create the final double stranded cDNA library, which is then purified and quantified by quantitative PCR. Each transcriptome library is sequenced on an Illumina NextSeq 500 as paired-end 75 bp reads or MiSeq as paired-end 75 bp and 150 bp.

The sequence reads were aligned to the UCSC hg19 reference genome using STAR (version 2.4.0; Dobin et al., 2013). After excluding read pairs with a mapping quality of <30, relapsed tumor-specific variants, which met the criteria of more than 10× total depth, 4× variant depth, and 10% variant frequency were called by VarScan2 (Koboldt et al., 2012). Gene expression analysis for RNA-seq was performed based on fragments per kilobase million using cufflinks (version 2.2.1; Trapnell et al., 2010). Enriched pathways between baseline and relapsed tumor of JFCR-119 were analyzed using gene set enrichment analysis (Subramanian et al., 2005), with a false discovery rate of (FDR) <0.25 and a P value of <0.05. Gene sets were downloaded from the Broad Institute’s MSigDB.

For targeted amplicon sequencing, the library was prepared using a Haloplex custom panel (Agilent), which is designed to detect well-known cancer-associated somatic mutations. Paired-end sequencing (2 × 150 bp) was performed on the MiSeq platform. Whole exon sequencing was performed by Cancer Precision Medicine Inc.

Sequence reads were aligned to the UCSC hg19 reference genome using Burrows–Wheeler Aligner (version 0.7.10; Li and Durbin, 2009). Read pairs with a mapping quality of <30 and with mismatches more than 5% of read length were excluded. Somatic variants were called by in-house pipeline (Kiyotani et al., 2017), with the criteria of more than 10× total depth, 4× variant depth, 10% variant frequency in tumor, less than 2% variant frequency in normal, and a Fisher’s P value of 0.05.

### Reverse transcription, gene cloning, and Sanger sequencing

cDNA was generated using a Transcriptor First Strand cDNA Synthesis Kit (Roche). To clone human PD-L1, nested PCR was performed using the primer for the first PCR: forward 5′-AGA​AAG​ATG​AGG​ATG​TTT​GCT​GTC-3′ and reverse 5′-TGG​TTA​CGT​CTC​CTC​CAA​ATG​TG-3′ and for the second PCR: forward 5′-CAC​CAT​GAG​GAT​ATT​TGC​TGT​CTT​TA-3′ and reverse 5′-TTA​CGT​CTC​CTC​CAA​ATG​TGT​ATC-3′. For human PD-1, PCR was conducted using forward 5′-CAC​CAT​GCA​GAT​CCC​ACA​GGC​GCC​CTG-3′ and reverse 5′-TCA​GAG​GGG​CCA​AGA​GCA​GTG​TCC-3′. For human TDP43, PCR was performed with forward 5′-CAC​CAT​GTC​TGA​ATA​TAT​TCG​GGT​AAC-3′ and reverse 5′- CTA​CAT​TCC​CCA​GCC​AGA​AGA​C-3′. For mouse PD-L1, PCR was conducted with forward 5′-CAC​CAT​GAG​GAT​ATT​TGC​TGG​CAT​TA-3′, reverse 5′- TTA​CGT​CTC​CTC​GAA​TTG​TGT​A-3′. PCR products were cloned into a pENTR vector according to the manufacturer’s protocol for the pENTR/D-TOPO Cloning Kit (Thermo Fisher Scientific). PD-L1ex4-7 (the genomic sequence of PD-L1 from exon 4 to the end of ORF) was cloned into a pcDNA3 vector (Thermo Fisher Scientific) using the following primers: forward 5′-AAA​AGA​ATT​CTG​TCC​TAG​CCC​CAT​ACA​ACA​AAA​TCA​ACC-3′ and reverse 5′-AAA​AAC​TCG​AGT​TAC​GTC​TCC​TCC​AAA​TGT​G-3′. To generate pENTR-mPD-L1v242, pENTR-mPD-L1v178, pENTR-hTDP43-W334L, pENTR-hTDP43-M337V, and pENTR-hTDP43-R361T, site-directed mutagenesis was performed using the following primers: for mPD-L1v242, forward 5′-AGG​ACT​CAC​TGG​TGA​GAA​TGC​TAG​ATG-3′ and reverse 5′-CTA​GCA​TTC​TCA​CCA​GTG​AGT​CCT​GTT​C-3′; for mPD-L1v178, forward 5′-CGT​GAG​TGG​AGA​TTA​AAG​CCA​GGG​CAA​AAC-3′ and reverse 5′-TTG​CCC​TGG​CTT​TAA​TCT​CCA​CTC​ACG​G-3′; for hTDP-43-W334L, forward 5′-AGA​GCA​GTT​TGG​GTA​TGA​TGG-3′ and reverse 5′-TCA​TAC​CCA​AAC​TGC​TCT​GTA​G-3′; for hTDP-43-M337V, forward 5′-GGG​GTA​TGG​TGG​GCA​TGT​TAG-3′ and reverse 5′-ACA​TGC​CCA​CCA​TAC​CCC​AAC-3′; and for hTDP-43-R361T, forward 5′-AAC​ATG​CAG​ACG​GAG​CCA​AAC-3′ and reverse 5′-TTT​GGC​TCC​GTC​TGC​ATG​TTG​C-3′. pLenti6.3 lentiviral vectors for expression were produced by LR cloning (Thermo Fisher Scientific).

To prepare human IgG1 Fc-tagged PD-L1 variants, PCR was performed using the Age I restriction site tagged forward primer 5′-AAA​AAA​AAA​AAC​CGG​TAT​GAG​GAT​ATT​TGC​TGT​C-3′ and the Xho I restriction site tagged reversed primer 5′-AAA​AAA​AAA​ACT​CGA​GCG​TCT​CCT​CCA​AAT​GTG​TA-3′ for PD-L1v229 and 5′-AAA​AAA​AAA​ACT​CGA​GTT​CTC​CCA​AGT​GAG​TCC​TTT​C-3′ for PD-L1v242. PCR products were cloned into pFUSE-hIgG1-Fc1 vector (InvivoGen). To correct the reading frame and generate PD-L1 (19–239), site-directed mutagenesis was performed using the following primers: forward 5′-TTG​GAG​GAG​ACG​TCG​AGC​ACC​ATG​GTT​AG-3′ and reverse 5′-CTA​ACC​ATG​GTG​CTC​GAC​GTC​TCC​TCC​AA-3′ for PD-L1v229; forward 5′-ACT​CAC​TTG​GGA​GAA​TCG​AGC​ACC​ATG​G-3′ and reverse 5′-CCA​TGG​TGC​TCG​ATT​CTC​CCA​AGT​GAG​T-3′ for PD-L1v242; and forward 5′-CCA​AAT​GAA​AGG​ACT​TCG​AGC​ACC​ATG​G-3′ and reverse 5′-CCA​TGG​TGC​TCG​AAG​TCC​TTT​CAT​TTG​G-3′ for PD-L1(19–239). Alterations by TDP-43 to the splicing in PD-L1 were detected by PCR using the following primers: forward 5′-ACC​ACC​ACC​AAT​TCC​AAG​AG-3′ and reverse 5′-TTA​CGT​CTC​CTC​CAA​ATG​TGT​ATC-3′.

Sanger sequencing was performed following the manufacturer’s protocol for the BigDye Terminator v3.1 Cycle Sequencing Kit (Thermo Fisher Scientific).

### Quantitative reverse transcription PCR analysis

To quantify the level of PD-L1 mRNA in the PD-L1 variant overexpressing cells, cDNA, Fast SYBR green Master Mix (Roche), and primers (PD-L1: forward 5′-TGG​CAT​TTG​CTG​ACG​CAT​TT-3′ and reverse 5′-TGC​AGC​CAG​GTC​TAA​TTG​TTT​T-3′; GAPDH: forward 5′-TGC​ACC​ACC​AAC​TGC​TTA​GC-3′ and reverse 5′-GGC​ATG​GAC​TGT​GGT​CAT​GAG-3′) were mixed. The reaction was performed using the LightCycler 96 system (Roche).

### PD-L1 splicing variant specific PCR

For precise detection of PD-L1v242, nested PCR was performed using the primer for first PCR: forward 5′-AGT​TCT​GCG​CAG​CTT​CCC​GAG-3′ and reverse 5′-CCC​TGC​TTG​AAG​ATC​AGA​AGT​TCC-3′. After purifying the PCR products following the manufacturer’s protocol for Ampure XP (Beckman Coulter), a second PCR was conducted using the primer: forward 5′-TGG​CAT​TTG​CTG​ACG​CAT​TT-3′ and reverse 5′-TGC​AGC​CAG​GTC​TAA​TTG​TTT​T-3′ for amplifying whole PD-L1 variants and forward 5′-AGG​ACT​CAC​TTG​GGA​G-3′ and reverse 5′-TTA​CGT​CTC​CTC​CAA​TCT​GTA​TCA-3′ for amplifying the PD-L1v242 variant.

### Cell lines

PC-9, SW480, Jurkat, THP-1, and Chinese hamster ovary (CHO) cell lines from ATCC were cultured in RPMI-1640 with 10% FBS, 100 units/ml penicillin, and 100 µg/ml streptomycin. MC38 was obtained from Kerafast and cultured in low-glucose DMEM supplemented with 10% FBS, 2 mM glutamine, 0.1 mM nonessential amino acids, 1 mM sodium pyruvate, 10 mM HEPES, 50 µg/ml gentamycin sulfate, 100 units/ml penicillin, and 100 µg/ml streptomycin. 293FT cells were cultured in high-glucose DMEM with 10% FBS.

### Transfection, lentiviral production, and lentiviral infection

For transient expression, pLenti6.3 or pFUSE-hIgG1-Fc1 vectors were transfected with Lipofectamine 2000 (Thermo Fisher Scientific) or FuGENE HD transfection reagent (Promega) for 24 h.

Lentivirus production was performed by cotransfecting the pLenti6.3 and helper plasmids (ViraPower) in 293FT cells for 24 h following the manufacturer’s protocol for ViraPower Lentiviral Expression Systems (Thermo Fisher Scientific).

Viruses were used for infection with 4 µg/ml polybrene. After 48 h infection, cells were selected using blasticidin at 5 µg/ml for MC38 and at 10 µg/ml for other cells for 5 d. To establish stable PD-L1 variants overexpressing PC-9 and SW480, the cells were cloned.

### Flow cytometry analysis

To evaluate the PD-L1 expression on the cell surface, 3 × 10^5^ cells were prepared in 50 µl FACS buffer (PBS with 0.5% BSA). 1 μl PE-conjugated aPD-L1 antibody (aPD-L1-PE, 29E.2A3; BioLegend), aPD-1-PE (EH12.2H7; BioLegend), or isotype control-PE (MOPC-21 or MG2b-57; BioLegend) was added and incubated for 30 min at 4°C.

To test the influence of the sPD-L1 splicing variant on the aPD-L1 or aPD-1 antibody, preincubated samples containing 125 µg/ml aPD-L1-PE or aPD-1-PE and the sPD-L1 splicing variant at the indicated molar ratio in 50 µl FACS buffer were mixed with 5 × 10^5^ PD-L1 aAPC/CHO-K1 or Jurkat/PD-1 cells for 1 h at 37°C. Following incubation for 30 min at 4°C, the staining was halted by washing with 400 µl FACS buffer. After removing the buffer, the cells were resuspended with 500 µl FACS buffer and evaluated with FACS Verse (BD Biosciences).

To test the binding of sPD-L1 splicing variants to PD-1, culture supernatant containing Fc-tagged sPD-L1 splicing variants was incubated with Jurkat/PD-1 or CHO/PD-1 cells for 1 h at 37°C. Following washing with 400 µl FACS buffer, the samples were incubated with Alexa Fluor 488 anti-human IgG (Invitrogen; 1:500) for 30 min at 4°C and washed once with FACS buffer. After removing the buffer, the cells were resuspended with 500 µl FACS buffer and assayed with FACS Verse (BD Biosciences). The data were analyzed using FlowJo software (TOMY Digital Biology).

### Enzyme-linked immunosorbent assay

Ni-NTA HisSorb Plates (Qiagen) were blocked with 280 µl ELISA buffer (10 mM Tris, pH 7.5,150 mM NaCl, 5 mM CaCl_2_, 0.1% BSA, and 0.05% Tween 20). Afterward, 100 ng of His-tag conjugated PD-1 (BPS Bioscience) was applied to the plates overnight at 4°C. The plates were washed once with 280 µl ELISA buffer. Human IgG1 Fc-tagged recombinant PD-L2 (PeproTech), human IgG1 Fc-tagged recombinant B7-H3 (ACRObiosystems), purified Fc-fusion sPD-L1 splicing variants, or human IgG1 Fc-tagged recombinant PD-L1 (Thermo Fisher Scientific), including blockade antibody and PD-L1v242, as described, were incubated in wells for 2 h at room temperature (RT). After the plates were washed once with 280 µl ELISA buffer, 100 µl of HRP conjugated anti-human IgG antibody (GE Healthcare; 1:1,000) was added and incubated for 1 h at RT. Following three washes with ELISA buffer, the plates were incubated for 10–30 min at RT with 100 µl of 1-Step Ultra TMB-ELISA Substrate Solution (Thermo Fisher Scientific). Afterward, 2 N H_2_SO_4_ was added to each well to stop the reaction and the absorbance for each well was measured at 450 nm.

Concentrations of human and mouse PD-L1 were determined with a Human PD-L1 ELISA Kit (ab214565, Abcam) and a Mouse PD-L1 DuoSet ELISA kit (DY1019-05, R&D Systems), respectively, according to the manufacturers’ protocols. Before use, the samples were subjected to ultracentrifugation at 100,000 *g* for 90 min at 4°C to remove microparticles, including exosomes.

### IHC

IHC staining was performed on representative tissue sections from formalin-fixed and paraffin-embedded tissue blocks using anti-human CD8 antibodies (C8/144B; Nichirei Biosciences), anti-human PD-1 antibodies (NAT105; Abcam), anti-human HLA class I-A, B, and C antibodies (EMR8-5; HoKudo), anti-human B2M antibodies (A0072; Dako), anti-mouse CD8 antibodies (EPR20305; Abcam), anti-mouse PD-1 antibodies (D7D5W; Cell Signaling Technology), and anti-mouse granzyme B antibodies (D6E9W; Cell Signaling Technology).

### Immunofluorescence staining

The cells were fixed with 10% formaldehyde for 30 min and permeabilized with buffer containing 0.3% Triton X-100 and 1× Blocking One solution (Nacalai Tesque) for 1 h at RT. The cells were labeled with aPD-L1 antibodies (E1J2J, Cell Signaling Technology; 22C3, Dako; and SP142, Spring Bioscience) in buffer containing 0.3% Triton X-100 and 1× Blocking One solution (Nacalai Tesque) overnight at 4°C. They were then labeled with goat anti-Mouse IgG (H+L) cross-adsorbed secondary antibody, Alexa Fluor 488 (A11001; Thermo Fisher Scientific) or goat anti-rabbit IgG (H+L) highly cross-adsorbed secondary antibody, Alexa Fluor 488 (A11034; Thermo Fisher Scientific) in buffer containing 0.3% Triton X-100 and 1× Blocking One solution (Nacalai Tesque) for 1 h at RT. The nuclei were stained with Hoechst 33342 (Thermo Fisher Scientific). The images were captured by a FLUOVIEW FV1000 laser scanning microscope (Olympus).

### Purification of sPD-L1 splicing variant proteins

Human IgG1 Fc-tagged sPD-L1 splicing variants from the culture supernatant of PD-L1 variant overexpressing CHO cells were purified with Protein G Sepharose 4 Fast Flow (GE Healthcare).

To enrich the sPD-L1 splicing variants, the variants overexpressing PC-9 and SW480 cells were seeded in 15-cm dishes. When the confluence reached 80–90%, the medium was replaced with serum-free, phenol red-free RPMI-1640 for 24 h. The supernatant was collected, and the debris was removed by centrifugation at 2,000 *g* for 5 min at 4°C, then at 10,000 *g* for 15 min at 4°C. sPD-L1 splicing variant proteins in the supernatant were enriched with Vivaspin 20 with a 10 kD Molecular Weight Cutoff (GE Healthcare). Finally, sPD-L1 variant-concentrated supernatant was centrifuged at 100,000 *g* for 90 min at 4°C to remove micro particles including exosomes.

For quantification of the concentrated or purified sPD-L1 splicing variants, FLAG-Avi-His-tagged PD-L1 (BPS Bioscience) and hIgG1-Fc-tagged recombinant human PD-L1 (Thermo Fisher Scientific) were used as the standard.

### Western blot analysis

To analyze the PD-L1 variant secretion, ∼4 × 10^5^ PC-9 or SW480 cells overexpressing each variant were cultured for 24 h. The culture supernatant was centrifuged at 20,000 *g* for 5 min at 4°C, followed by 100,000 *g* for 90 min at 4°C to remove the debris and micro particles, including exosomes. Three times the volume of cold acetone was added, and the proteins in the supernatant were precipitated for over 2 h at −20°C. After centrifuging at 10,000 *g* for 10 min at 4°C, precipitated proteins were dried and resuspended in SDS lysis buffer (100 mM Tris, 1% SDS, 10% glycerol, and 10% 2-mercaptoethanol).

Western blotting was performed as previously described (Uchibori et al., 2017). Primary antibodies for PD-L1 (Cell Signaling Technology, 15165, 1:1,000; Cell Signaling Technology, 13684, 1:4,000; Abcam, ab205921, 1:2,000; Dako, SK006, 1:200; Spring, M4424, 1:1,000), HSP90 (Cell Signaling Technology, 4875, 1:1,000), and GAPDH (Millipore, MAB374, 1:5,000) were used.

### Immunoprecipitation

Cells were lysed in lysis buffer (20 mM Tris, pH 7.5, 1% NP-40) supplemented with protease inhibitors (Complete Mini; Roche) at 4°C for 30 min and were centrifuged to remove debris. aPD-L1 antibody (29E.2A3; BioLegend) was added to the cell lysates and culture supernatant containing protease inhibitors and incubated at 4°C overnight. After incubation with protein G mag sepharose (GE Healthcare) at 4°C for 1 h, the magnetic beads were washed three times with wash buffer (20 mM Tris, pH 7.5, 0.2% NP-40, 10% glycerol, 137 mM NaCl, 1.5 mM MgCl_2_, and 1 mM EDTA). Finally, the proteins were eluted from the magnetic beads using 2 × SDS lysis buffer heated at 100°C for 5 min.

### Protein half-life assays

PC-9 cells (5 × 10^5^) overexpressing sPD-L1 splicing variants were seeded into 6-well plates. The next day, the cells were treated with 100 µM cycloheximide (Sigma) and then harvested and lysed with cell lysis buffer containing protease inhibitors (20 mM Tris, pH 7.5, 1% NP-40) at the indicated time point. Whole-cell lysates (WCLs) and the culture supernatant were immunoprecipitated with aPD-L1 antibody (29E.2A3; BioLegend) and immunoblotted with another aPD-L1 antibody (E1J2J; Cell Signaling Technology).

### Pulse-chase assays

PC-9 cells (10^7^) overexpressing sPD-L1 splicing variants were seeded in 100-mm dishes. After overnight culture, the medium was changed to methionine-free RPMI-1640 (Sigma) containing 10% FBS and cultured for 2 h at 37°C. Afterward, 50 µCi of ^35^S-labeled methionine (PerkinElmer) was added to the medium. After 2 h culture at 37°C, the medium was replaced with serum-free RPMI-1640 containing 1 mM label free methionine. Cells and culture supernatant were harvested at the indicated time point, and the samples were immunoprecipitated with aPD-L1 antibody (29E.2A3; BioLegend) and SDS-PAGE. The cells were visualized with a phosphor imaging scanner (Typhoon 9410; GE Healthcare). The band intensity was quantified using ImageJ software (National Institutes of Health).

### Glycosylation analysis

To analyze the glycosylation status, the sPD-L1 splicing variant-concentrated supernatant and the WCL of PC-9 or PC-9/PD-L1 lysed with lysis buffer (50 mM Tris, pH 8, 150 mM NaCl, 5 mM EDTA, 1% NP-40, and 0.1% SDS) were prepared. O-glycanase, N-glycanase, and sialidase-A treatment were performed according to the denaturing protocol for the GlycoPro Enzymatic Deglycosylation Kit (PROzyme).

### PD-1/PD-L1 blockade bioassay

The assay was performed by following the manufacturer’s protocol for the PD-1/PD-L1 blockade bioassay (Promega).

In brief, 4 × 10^5^ aAPC/CHO-K1 or PD-L1 aAPC/CHO-K1 cells were seeded into 96-well plates in RPMI-1640 with 10% FBS. After overnight culturing, the medium was aspirated, and then aPD-L1 or aPD-1 blockade antibody as indicated and 5 × 10^5^ PD-1 effector cells were added. For assessing the sPD-L1 splicing variants trapping the antibody, preincubated samples containing aPD-L1 or aPD-1 blockade antibody and sPD-L1 splicing variants were mixed at the indicated mole ratio for 1 h at 37°C and were cocultured with 5 × 10^5^ PD-1 effector cells for 6 h. Following mixing with Bio-Glo Reagent, the luminescence was measured with a luminescence plate reader (TriStar LB 941; Berthold).

### Apoptosis induction assay by the coculture of T cells overexpressing PD-1 and antigen-presenting cells overexpressing PD-L1

WT-1 antigen-specific T cells were regenerated from iPSCs that had been originally derived from WT-1 antigen-specific T cells, as previously described (Maeda et al., 2016). The regenerated T cells were then lentivirally transduced with PD-1 gene. Approximately 80% of regenerated T cells expressed PD-1 (reT/PD-1). THP-1 (human leukemic cell line) cells, which express endogenous WT-1 antigen, were transduced with PD-L1 gene and maintained with blasticidin for selection of stable transfectants (THP-1/PD-L1). THP-1 or THP-1/PD-L1 cells (3 × 10^4^) were cocultured with reT/PD-1 cells (3 × 10^4^) in 96-well V-bottom plate in the presence of aPD-L1 antibody (1 µg/ml) or PD-L1v242 (2 µg/ml) for 18 h at 37°C. Ratio of dead cells in reT/PD-1 cells was flow cytometrically measured using propidium iodide staining.

### In vivo experiment using MC38 syngeneic mice model

All mice studies were conducted in line with the protocols approved by the Committee for the Use and Care of Experimental Animals of the Japanese Foundation for Cancer Research.

10^6^ MC38, MC38/cont, MC38/mPD-L1, MC38/mPD-L1v242, and MC38/mPD-L1v178 tumor cells in 50 µl HBSS were subcutaneously injected into the right flank of 6-wk-old C57BL/6 female mice (Charles River). When the average estimated tumor volume was around 100∼200 mm^3^, the mice were grouped randomly with comparable tumor volume. The treatment of control IgG (Sigma), aPD-L1 antibody (10F.9G2; BioLegend), and aPD-L1 antibody (RMP-1-14; BioLegend) was conducted intraperitoneally three times per week, and the tumor volume was calculated as length × width^2^ × 0.5 (mm^3^).

### Statistical analysis

Paired two-tailed Student *t* test was used for the cell culture experiments. Kaplan-Meier curves were evaluated using the Gehan–Breslow–Wilcoxon test. P < 0.05 was considered to be significant. *, P < 0.05; **, P < 0.01; ***, P < 0.001.

### Online supplemental material

Fig. S1 shows gene analysis of the tumor that was relapsed from PD-L1 blockade therapy. Fig. S2 shows a schematic diagram of PD-L1 C-terminal deficient splicing variants. Fig. S3 shows sPD-L1 splicing variants prevent NFAT reactivation by PD-L1 blockade but not by PD-1 blockade. Fig. S4 shows a schematic illustration of similarity between mouse and human sPD-L1 variants. Table S1 shows information of clinical samples and targeted amplicon sequencing.
